# QTLTableMiner^++^: semantic mining of QTL tables in scientific articles

**DOI:** 10.1186/s12859-018-2165-7

**Published:** 2018-05-25

**Authors:** Gurnoor Singh, Arnold Kuzniar, Erik M. van Mulligen, Anand Gavai, Christian W. Bachem, Richard G.F. Visser, Richard Finkers

**Affiliations:** 1 Plant Breeding, Wageningen University and Research, Wageningen, The Netherlands; 2 Netherlands eScience Center (NLeSC), Amsterdam, The Netherlands; 3 Department of Medical Informatics, Erasmus Medical Center, Rotterdam, The Netherlands

**Keywords:** Quantitative trait locus, QTL, Plant breeding, Table mining, Ontologies, Semantic interoperability

## Abstract

**Background:**

A quantitative trait locus (QTL) is a genomic region that correlates with a phenotype. Most of the experimental information about QTL mapping studies is described in tables of scientific publications. Traditional text mining techniques aim to extract information from unstructured text rather than from tables. We present QTLTableMiner^++^ (QTM), a table mining tool that extracts and semantically annotates QTL information buried in (heterogeneous) tables of plant science literature.

QTM is a command line tool written in the Java programming language. This tool takes scientific articles from the Europe PMC repository as input, extracts QTL tables using keyword matching and ontology-based concept identification. The tables are further normalized using rules derived from table properties such as captions, column headers and table footers. Furthermore, table columns are classified into three categories namely column descriptors, properties and values based on column headers and data types of cell entries. Abbreviations found in the tables are expanded using the Schwartz and Hearst algorithm. Finally, the content of QTL tables is semantically enriched with domain-specific ontologies (e.g. Crop Ontology, Plant Ontology and Trait Ontology) using the Apache Solr search platform and the results are stored in a relational database and a text file.

**Results:**

The performance of the QTM tool was assessed by precision and recall based on the information retrieved from two manually annotated corpora of open access articles, i.e. QTL mapping studies in tomato (*Solanum lycopersicum*) and in potato (*S. tuberosum*). In summary, QTM detected QTL statements in tomato with 74.53% precision and 92.56% recall and in potato with 82.82% precision and 98.94% recall.

**Conclusion:**

QTM is a unique tool that aids in providing QTL information in machine-readable and semantically interoperable formats.

**Electronic supplementary material:**

The online version of this article (10.1186/s12859-018-2165-7) contains supplementary material, which is available to authorized users.

## Background

Modern genetic analysis in crop plants aims to understand the contribution of individual genes and loci in the development of agronomic traits. Quantitative variation results from the combined action of multiple genes and environmental factors. With the help of molecular marker studies, it is possible to detect genomic regions that are statistically associated with variation in non-Mendelian phenotypic traits, also termed as quantitative trait loci (QTL) [1].

Detecting QTLs can help in the development of precision breeding programs. However, elucidating QTL regions for genes that are causative to a trait of interest is a tedious and time-consuming process because a single QTL region commonly entails hundreds of genes, including those that might negatively influence the trait [2]. Leveraging upon knowledge available in both scientific literature and molecular biology databases can help in narrowing down the QTL regions to candidate genes associated with traits of interest.

QTL studies have widely been published in scientific articles, in particular in tables or supplementary materials. However, there is no established repository where experimental data on plant-specific QTL studies can be submitted. In the past, there have been several attempts to create manually curated databases with QTL information; for example, AnimalQTLdb [3], MaizeGDB [4], Gramene QTL database [5] and SGN/solQTL [6]. Manual curation of such database systems is a laborious task. There is a need to retrieve QTL data from publications efficiently, which can further reduce the cost of QTL database curation and QTL knowledge discovery process.

Using tables is the most common way to represent (semi-)structured data (e.g. results of QTL mapping experiments) on the web or in the scientific literature [7]. As QTL information is mostly published in tables rather than in the main text of articles, traditional text-mining techniques are not suited for this task [8]. There are several challenges associated with table-mining. The information in a table can be easily interpreted by human but not by a machine. For example, when parsing an article in text, HTML or PDF formats, it is difficult for a machine to determine which cells are part of a header and which cells contain data. Moreover, tables can have different orientations (horizontal *versus* vertical layout). Furthermore, tables can have nested structure including rows/columns with multiple headers.

Several commercial and open source table-mining tools have been developed including Tabula [9], Google Tables [10, 11], TableMiner^+^ [12] and the domain-specific QTLMiner [8]. While Tabula and QTLMiner extract tables from PDF documents, Google Tables and TableMiner^+^ process web pages. TableMiner^+^ makes use of contextual information, for example, in table captions, footers and column headers, to improve the identification of relevant tables in web pages. In contrast, the Google’s system does not use author-defined table properties, such as column headers, captions and footers, but rather assigns class-labels to columns using a machine-learning approach combined with maximum likelihood estimation over web-derived knowledgebase. QTLMiner [8] was the first tool focused on mining QTLs from tables of plant science literature. Briefly, QTLMiner first converts articles in PDF to HTML documents, identifies trait-related tables, extracts relevant data and finally stores the results in a relational database. QTLMiner lacks wider applicability as its performance to extract information from tables of a literature, depends mainly on the conversion of articles from PDF to HTML file, which is done by commercially available web service from BCL [13]. Secondly, QTLMiner could only extract QTL statements only when a table in literature occur in a particular format and lacks the capability to mine this information from heterogeneous tables.

Current tools that extract tabulated information from PDF or HTML documents have difficulty with parsing tables correctly because table structures are (semantically) not described using these formats. Although, scientific articles are distributed in PDF format, it is inconvenient to use these PDF documents for automated information extraction as they lack machine readability and a logical structure specifying which content constitutes a paragraph, table, figure, header or footer etc. Therefore, even if massive amounts of unstructured data are held in the form of PDF documents, automated extraction of tables, figures or other structured information can be very difficult. Similarly, HTML file represents a layout of a web page and is not focused on describing data. Therefore, our tool uses XML files as they represent information in a logical structure that is machine-readable.

QTLTableMiner^++^ (QTM) is a Java-based command-line tool that extracts and semantically annotates QTL information from tables of scientific articles. QTM takes articles in a syntactically interoperable format, XML, as an input. The Europe PMC [14] repository provides full-text open access articles in the XML format that complies to the Journal Article Tag Suite (JATS) schema. JATS is commonly used by publishers and archives to exchange journal content.

QTM filters (candidate) trait tables (i.e. those with phenotypic information) out of all tables in an article. In these tables, a QTL statement refers to a relationship between pheno- and genotypic entities. QTM extracts QTL statements and semantically annotates the biological entities in these statements with domain-specific ontologies using the Apache Solr search platform [15]. Finally, QTM outputs the results both in a relational database and in a text file (CSV). In summary, QTM is a unique tool that aids in providing QTL information in machine-readable and semantically interoperable formats.

## Implementation

### Table mining workflow

**Fig. 1:**
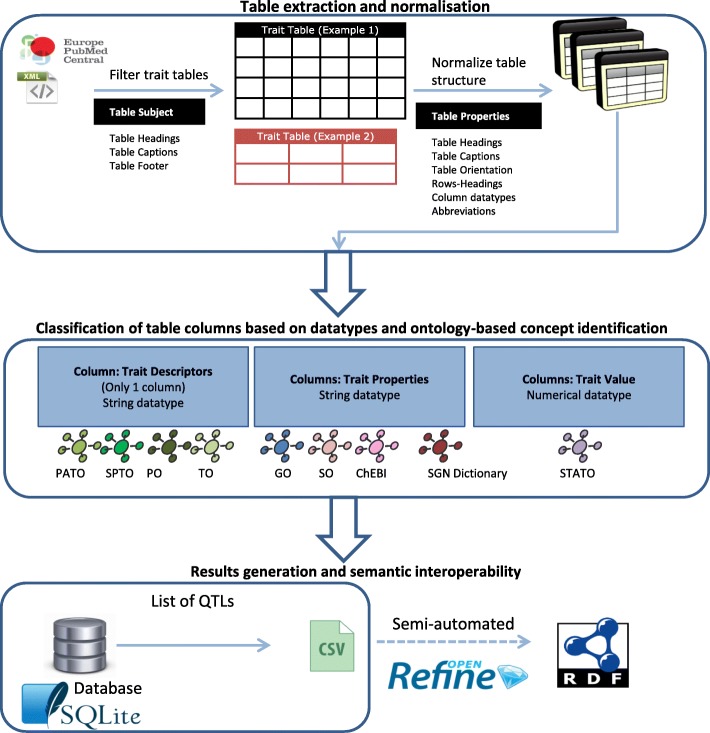
QTLTableMiner^++^ workflow including semantic transformation using OpenRefine

Figure 1 illustrates the overall workflow implemented by the QTM tool. This workflow consists of three parts, which are described in more details below.

#### Table extraction and normalization

First, the QTM tool retrieves open access articles in *XML* format from the Europe PMC repository [14] using the programmatic web interface (RESTful API). Then it detects tables in the articles using the <table-wrap>.. </table-wrap> XML tags and filters trait-related tables using keyword matching against table subjects derived from captions, headings and footers.

**Fig. 2:**
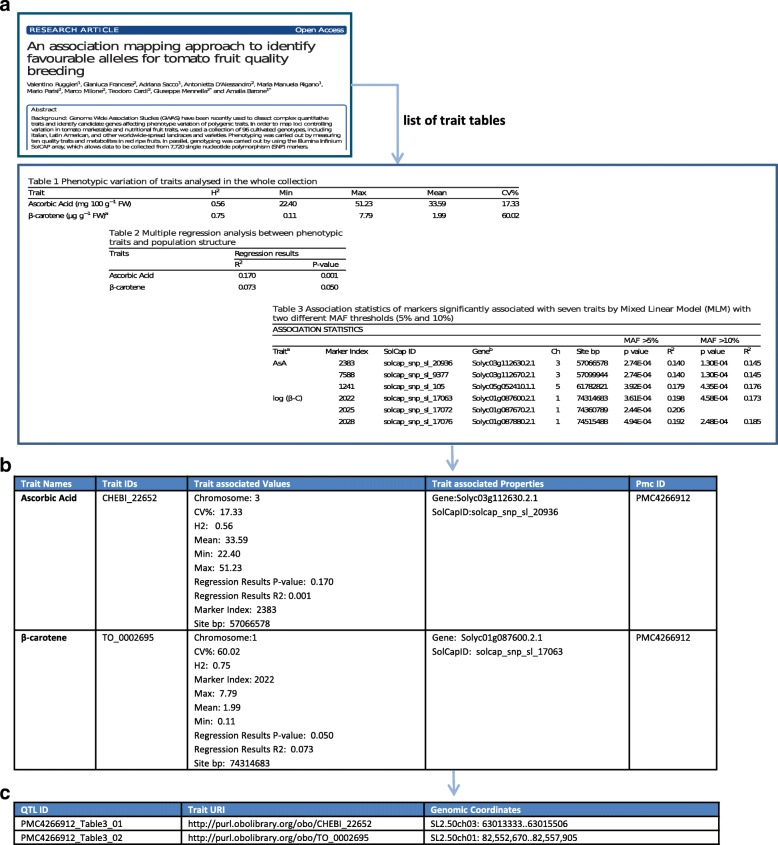
QTLTableMiner^++^ workflow exemplified on an article. **a** Input article (http://identifiers.org/pmc/ PMC4266912PMC4266912) with three trait tables (*Table 1-3*, only the top-two rows *per* table are shown), **b** trait statements identified in these tables, **c** output list of QTL statements

Tables are usually heterogeneous in structure (Fig. 2a). For example, they can have horizontal/vertical orientation, nested structure or headings that refer to more than one row or column. Although the XML output includes tables in (semi-)structured forms, further normalization of the tables is required to query over them. Therefore, we developed normalization rules based on table properties (e.g. captions, footers, column headers, data types and abbreviations). We use the Schwartz and Hearst abbreviation-expansion (S & H) algorithm to identify and expand all abbreviations found in table headings and cell entries [16].

After the normalization step, each table has a single row of headings including expanded abbreviations and each cell is identified by a pair of row/column indices.

#### Ontology-based concept identification and classification of table columns

QTM uses the Apache Solr search platform (version 6.2.1, [15]) to semantically annotate biological entities and statistical concepts found in tables using domain-specific dictionaries or ontologies. In particular, the Solanaceae Phenotype Ontology (SPTO) [17, 18], Plant Ontology (PO) [19, 20], Phenotypic Quality Ontology (PATO) [21, 22] and Trait Ontology (TO) [23] were used to identify plant-specific phenotypic information whereas Gene Ontology (GO) [24] and Sequence Ontology (SO) [25] were used to identify genotypic information. Further, small chemical compounds were annotated using the Chemical Entities of Biological Interest database/ontology (ChEBI) [26, 27]. Plant-specific genetic markers and gene or transcript IDs were obtained from the Sol Genomics Network (SGN) [28, 29]. STATistics Ontology (STATO) [30] was used to annotate the quantitative (statistical) results of QTL mapping experiments.

Table columns were classified according to the column properties into three categories: i) trait descriptors refer to a trait, phenotype or QTL in the column headings with alphanumeric data type (using SPTO, PO, PATO and TO); ii) trait properties refer to chemical compounds, genes, transcripts or genetic markers in all other columns with alphanumeric data type (using ChEBI, GO and SO); and iii) trait values are columns that contain exclusively numerical data types (using STATO).

#### Results generation and semantic interoperability

The last steps of the workflow involve extracting QTL statements from the trait tables and writing the annotated results into a relational database (SQLite v3.11.0) [31]. The database schema consists of six tables: *ARTICLE*, *TRAIT_TABLE*, *ABBREVIATION*, *QTL*, *COLUMN_ENTRY* and *CELL_ENTRY* (see Additional file 1: Supplementary Methods). In addition, the results stored in the *QTL* table are written into a text file (CSV).

Furthermore, the extracted QTL data were transformed into semantically interoperable RDF-based formats using the OpenRefine software [32]. The resulting RDF graphs including the SQLite database and CSV files were deposited at the Zenodo repository according to the FAIR (Findable, Accessible, Interoperable and Re-usable) Data guiding principles [33] (doi:10.5281/zenodo.1215044, [34]).

### Performance evaluation and validation

#### Experimental design

We assessed the performance of the QTM tool using two manually annotated corpora of 30 open access articles each. The first set contains QTL mapping studies of tomato (*Solanum lycopersicum*) whereas the second set is focused on potato (*Solanum tuberosum*). Although the presented version of the tool uses a tomato-specific dictionary to annotate genes, transcripts and genetic markers, it can be adopted for use on other crop species. QTM is expected to detect and semantically annotate biological entities such as genes and markers in the set ‘tomato’. However, QTM can also perform well on other species. For this, we use the second set of articles, i.e. set ‘potato’, for which QTM is expected to detect QTL statements without annotating biological entities such as genes and markers.

By our manual curation, the set ‘tomato’ included 66 trait tables with 2326 rows, 292 abbreviations, 757 biological entities and 405 QTL statements whereas the set ‘potato’ included 71 trait tables with 1292 rows, 207 abbreviations, 200 biological entities and 196 QTL statements. Specifically, precision and recall measures were obtained at four distinct levels of i) trait table, ii) abbreviation, iii) biological entities, and iv) QTL statement. Each result set was classified into four disjoint classes of the confusion matrix (i.e. true positives (TP), false positives (FP), true negatives (TN) and false negatives (FN)). Precision and recall were calculated as TP / (TP + FP) and TP / (TP + FN), respectively.

#### Runtime and memory usage

The runtime and memory usage of the QTM tool were collected using three sets of articles (*N*=10, 20 or 30) derived from the tomato-specific corpus.

## Results

### Workflow demonstration on exemplary articles

QTM takes one or more PubMed Central identifiers (PMCIDs) as input and returns a list of QTL statements, further exemplified by an article (PMC4266912) in Fig. 2. In this article, there are three trait tables (i.e. Tables 1, 2 and 3) with a total 35 rows out of which only 8 were QTL statements (Table 3). The tool detected 7 out of 8 QTL statements.

In each QTL statement, biological entities such as traits, genes and markers were annotated using ontological terms. In the 7 QTL statements detected, there were 7 unique traits linked to 7 genes and 7 SNP-based markers. In particular, QTM annotated a subset of traits (3 out of 7) while it detected all genes and markers (7 out of 7).

Importantly, QTL statements from multiple tables can be combined using the ontology-based annotation and the S & H abbreviation-expansion algorithm. For example, the statements including terms such as *ascorbic acid* (CHEBI:22652) and *β*-*carotene* (TO:000269) were combined from the three tables (Fig. 2b). Note that both terms were abbreviated as *AsA* and *β*-C in this article (*Table 3*). Finally, QTM outputs all QTL statements detected in the article(s) (Fig. 2c).

### Performance evaluation on both tomato and potato datasets

#### Detection of trait tables

**Fig. 3:**
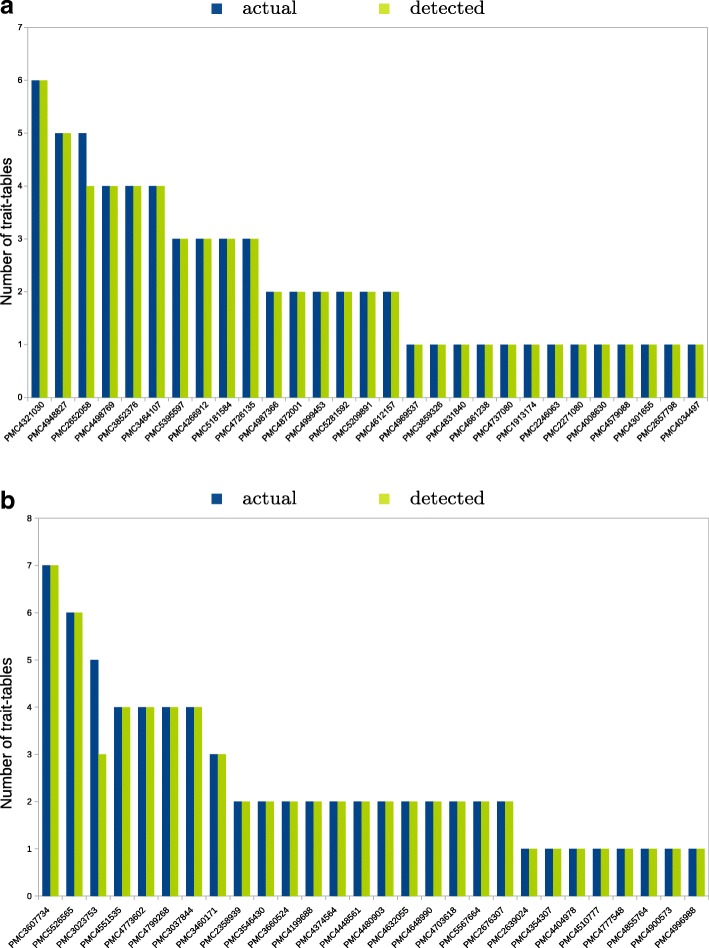
Bar graphs of the numbers of QTL tables detected per article for the manually curated set ‘tomato’ (**a**) and set ‘potato’ (**b**) using the QTLTableMiner^++^

The QTM tool recovered almost all trait-related tables for both manually curated corpora (Fig. 3). All trait tables were correctly identified except Table 1 of PMC2652058 (in tomato) and Tables 1 and 2 of PMC3023753 (in potato). In fact, these three tables eluded detection due to missing words such as trait, QTL or phenotype in their descriptions and/or bodies.

The detection of trait tables reached 100% precision for both sets whereas the recall was slightly lower (98.48% for tomato and 97.18% for potato).

#### Detection of trait-specific abbreviations

Detecting abbreviations is a prerequisite for reliable annotation of biological entities (e.g. traits, genes and markers) using standardized terms from domain-specific dictionaries or ontologies.

**Fig. 4:**
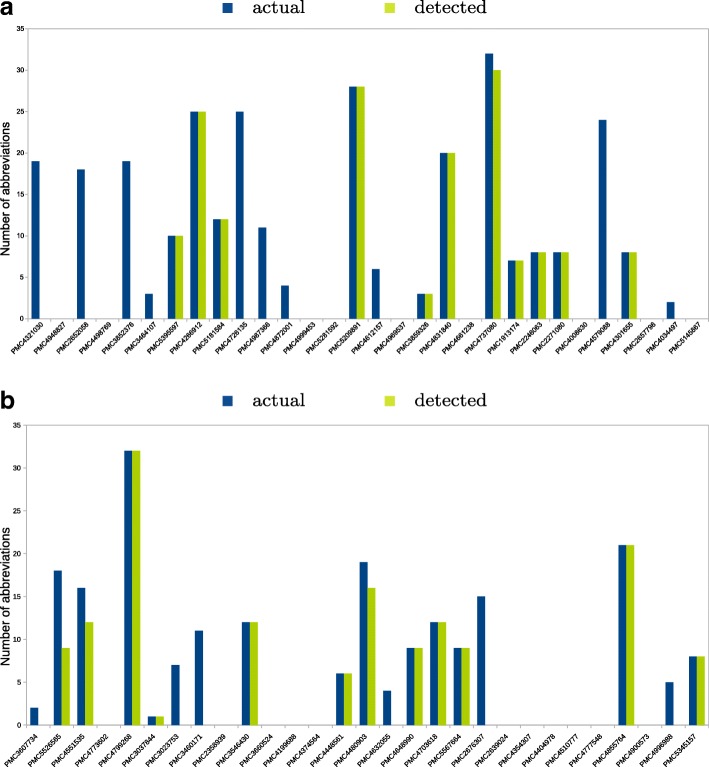
Bar graphs of the numbers of abbreviations detected per article for the manually curated set ‘tomato’ (**a**) and set ‘potato’ (**b**) using the QTLTableMiner^++^

QTM detected abbreviations in the trait tables found in 10 out of 20 articles in set ‘tomato’ and in 12 out of 19 articles in the set ‘potato’ (Fig. 4). As the S & H algorithm is a rule-based approach, QTM performs in all or nothing (binary) manner. This means that if the statements mentioning abbreviations were written in the algorithm required formations (long form (abbreviation) or abbreviation (long form)), QTM was able to detect all the abbreviations and *vice versa*.

QTM identified 159 out of 292 abbreviations (recall of 54.45%) for tomato and 147 out of 207 abbreviations (recall of 71.01%) for potato in the trait tables. All abbreviations were true positives (100% precision).

#### Annotation of biological entities

**Fig. 5:**
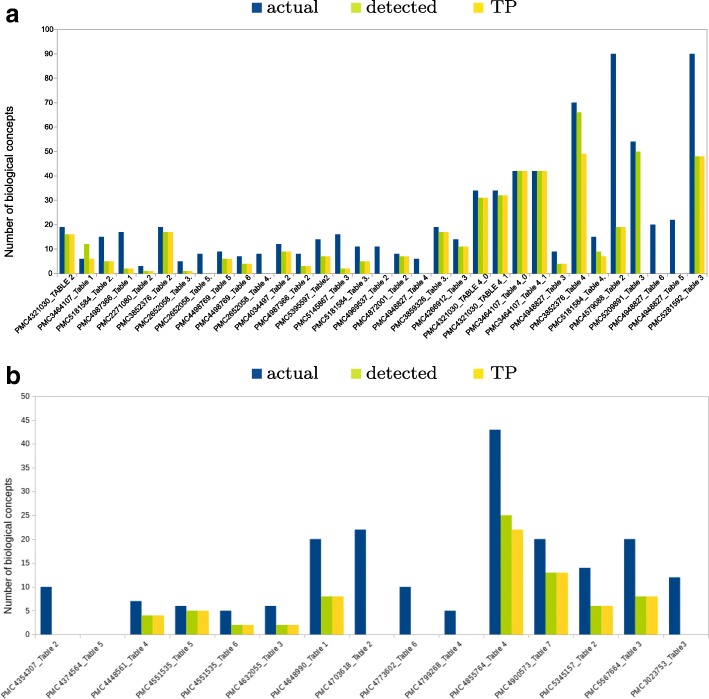
Bar graphs of the numbers of biological entities detected in trait tables for the manually curated set ‘tomato’ (**a**) and set ‘potato’ (**b**) using the QTLTableMiner^++^

QTM identifies and semantically annotates biological entities such as genes, genetic markers, proteins, metabolites or traits. In the set ‘tomato’, QTM detected 468 out of 757 biological entities, of which 393 were TP, 82 were FP, and 288 were FN with a recall of 57.71% and a precision of 82.74%. Similarly, in the set ‘potato’ QTM detected 73 biological entities out of the total 200. There were a total of 62 TP, 3 FP, 127 were FN. Here, the recall was low (35.53%) but the precision was high (95.89%). These results are shown in the Fig. 5.

#### Detection of QTL statements

**Fig. 6:**
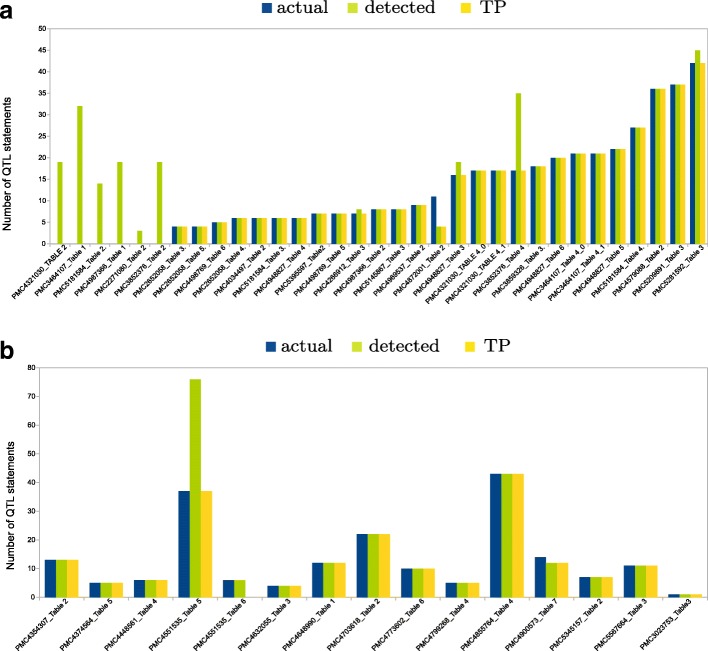
Bar graphs of the numbers of QTL statements detected in trait tables for the manually curated set ‘tomato’ (**a**) and set ‘potato’ (**b**) using the QTLTableMiner^++^

The main objective of QTM is to find QTL statements in tables. In the set ‘tomato’, QTM detected 529 QTL statements while the actual number of QTL statements were only 405. There were a total of 398 TP, 136 FP and 32 FN. Here, there is an increase in the number of FP statements detected due to the fact that QTM has difficulties in dealing with columns with special characters. For example, in Table 1 of PMC4987366, QTM reads column Genotype as a column with alphanumeric data type due to the presence of characters ‘**’, and thereby associates traits with the given genotype. Nevertheless, QTM performed with a precision of 74.53% and recall of 92.56% in set ‘tomato’. Similarly, in the set ‘potato’ QTM detected 233 QTL statements while the actual number of QTL statements were total 196. There were a total of 188 TP, 39 FP and 2 FN, thus QTM performed with a high precision of 82.82% and a recall of 98.94%. These results are shown in the Fig. 6.

Table 1 tabulates the precision and recall obtained for each task described above. Furthermore, a detailed confusion matrices of set ‘tomato’ and set ‘potato’ are provided in Additional files 2 and 3 of the supplementary material respectively.

**Table 1:** Benchmark results of the QTLTableMiner^++^ tool on different tasks

Detection	Precision (%)		Recall (%)	
	Tomato	Potato	Tomato	Potato
QTL tables	100	100	98.55	97.18
Abbreviations	100	100	54.45	71.01
Biological entities	82.74	95.89	57.71	35.53
QTL statements	74.53	82.82	92.56	98.94

#### Runtime and memory use

Table 2 summarizes the runtime and memory use of the QTM tool for three sets of full-text articles (using a commodity hardware with Intel Core i5 CPU, 4 GB RAM, 228 GB SSD, Ubuntu Linux 16.04.3 LTS). The results indicate that both the runtime and memory use increase approx. linearly with the amount of input.

**Table 2:** Scalability of the QTLTableMiner^++^ tool in terms of runtime and memory use

Number of articles	Number of tables	Number of rows in tables	Runtime (HH:MM:SS)	Max. memory (MB)
10	42	1562	00:04:10	19
20	58	2090	00:06:56	23
30	66	2326	00:07:58	30

## Discussion

QTM extracts QTL statements from tables of scientific articles as well as enables (re)publishing these statements in machine-readable and semantically interoperable RDF-based formats. Although it is possible to include review papers as an input for this tool, more accurate information can be obtained in the primary-data papers. Review papers frequently contain abbreviated references to the original papers and not the primary data.

Although, this tool was used to extract trait tables from plant-specific literature, the approach is also applicable to other domains. For example, a similar approach was used by Mulwad et al. [35] and Milosevic et al. [36] to retrieve health-related indicators about patients (e.g. the body mass index or BMI) from clinical literature. An important component in the QTM workflow is the use of the RESTful API of the Europe PMC, which provides open access articles in the (semi-)structured XML format. The resulting XML output complies with the JATS schema, which is a *de facto* standard for archiving and interchanging scientific articles. One drawback of using Europe PMC is that it is mainly focused on the biomedical literature while the plant literature is not covered extensively in this repository. As a result, we had to restrict our input set of articles (60 in total). Recently, publishers such as Springer or Elsevier have released Web APIs, which provide access to articles in JATS-compliant XML format. Therefore, our tool can be extended to use these APIs in the near future.

In total, QTM detected 529 QTL statements associated with 73 traits in tomato and 233 QTL statements associated with 16 traits in potato. In the set ‘tomato’ the five most common traits associated with the detected QTL statements were pH (SP:0000170), fruit shape (SP:0000038), compound leaf (SP:0000177), fruit (SP:00000378), and stem (SP:0000193). Whereas, in the set ‘potato’ the five most common traits associated with the detected QTL statements were anthocyanin content (SP:0000016), fruit shape (SP:0000038), fructose content (SP:0000386), stem strength (TO:0000051), and plant fresh weight (TO:0000442).

QTM performed better in the detection of biological entities for the set ‘tomato’ in comparison to the set ‘potato’ because the dictionaries used to annotate genes and genetic markers were tomato specific. The QTM algorithm has two distinctive features: i) the classification of table columns according to column properties into trait descriptors, trait properties and trait values, as well as ii) the ontology-based concept identification and annotation. We also present an approach to transform the extracted QTL information into the form of triples (<*subject*> <*predicate*> <*object*>), where <*subject*> refers to a trait descriptor, <*predicate*> is the column heading and <*object*> refers to the cell value in that column. QTM outputs a list of QTL statements both in a CSV file and in a SQLite database. Using the Linked Data approach, the resulting QTL statements can be integrated with genome-sequencing and annotation data to develop new or improve upon existing precision breeding programs. Combining the information available in scientific literature and molecular biology databases will help in narrowing down the QTL regions to detect candidate genes associated with traits of interest.

## Conclusions

QTM is a tool that aids in extracting QTLs from literature and in sharing these valuable data assets in machine-readable and semantically interoperable formats, and as such can help in formulating strategies for breeding crops of interest.

## Availability and requirements

**Project name:** candYgene

**Tool name:** QTLTableMiner^++^

**Source code availability:** https://github.com/candygene/QTM

**User’s guide:** https://github.com/candygene/QTM/blob/master/README.md

**Supporting Data and Results:** 10.5281/zenodo.1215044

**Operating system:** Ubuntu Linux (Ubuntu 16.04.3 LTS)

**Programming language:** Java

**Other requirements:** Java 1.7, SQLite 3.x, Apache Solr 6.x

**Licence:** Apache License Version 2.0

## Additional files

Additional file 1 Entity-Relation (ER) diagram. ER diagram of the QTM database. (PDF 75 kb)

Additional file 2 QTM results including precision and recall for 30 articles of set ‘tomato’. (PDF 53 kb)

Additional file 3 QTM results including precision and recall for 30 articles of set ‘potato’. (PDF 44 kb)

## Abbreviations

*β*-C: *β-carotene*
API: Application programming interface
AsA: Ascorbic acid
ChEBI: Chemical entities of biological interest
FAIR: Findable, accessible, interoperable and re-usable
FN: False negatives
FP: False positives
GO: Gene ontology
JATS: Journal article tag suite
PATO: Phenotypic quality ontology
PMCIDs: PubMed central identifiers
PO: Plant ontology
S & H algorithm: Schwartz and Hearst abbreviation-expansion algorithm
SO: Sequence ontology
SGN: Sol genomics network
SPTO: Solanaceae phenotype ontology
STATO: STATistics ontology
TN: True negatives
TO: trait ontology
TP: True positives
QTL: Quantitative trait loci
QTM: QTLTableMiner^++^
