# Supplementary figures and images for: QTLTableMiner++: semantic mining of QTL tables in scientific articles
Source: BMC Bioinformatics. 2018 May 25;19:183. doi: 10.1186/s12859-018-2165-7 (PMC5970438; doi:10.1186/s12859-018-2165-7)

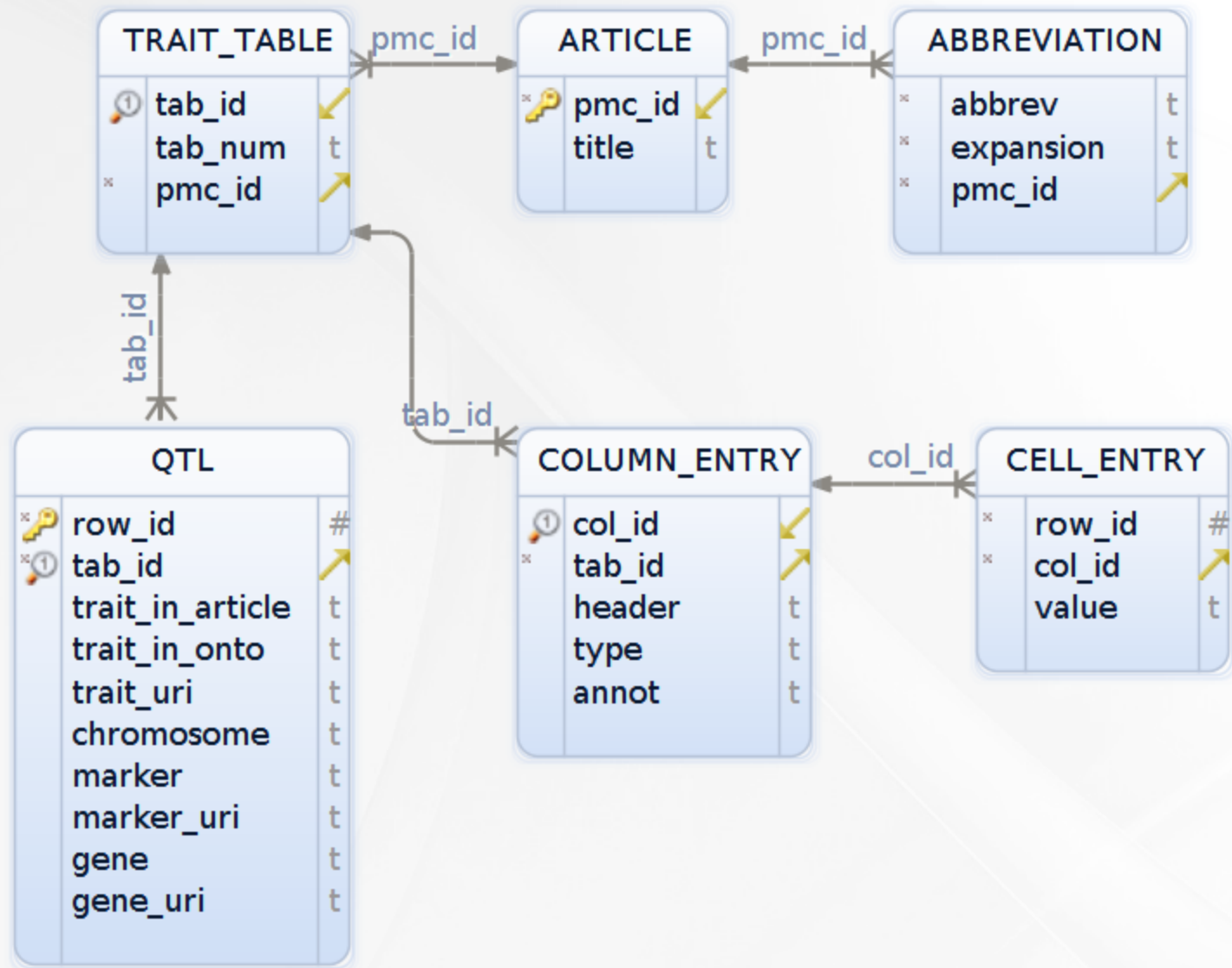

Supplement: Supplementary file 1 — Entity-Relation (ER) diagram. ER diagram of the QTM database. (PDF 75 kb) [file 12859_2018_2165_MOESM1_ESM.pdf]
